# Experimental and Theoretical Study of the Covalent Grafting of Triazole Layer onto the Gold Surface

**DOI:** 10.3390/ma13132927

**Published:** 2020-06-30

**Authors:** Nimet Orqusha, Sereilakhena Phal, Avni Berisha, Solomon Tesfalidet

**Affiliations:** 1 Department of Chemistry, University of Prishtina “Hasan Prishtina”, FNMS, str. “Nëna Tereze” nr. 5, 10000 Prishtina, Kosovo; nimet.orqusha@uni-pr.edu; 2 Department of Chemistry, Faculty of Science and Technology, Umeå University, SE 90187 Umeå, Sweden; sereilakhena.phal@umu.se; 3 Materials Science—Nanochemistry Research Group, NanoAlb—Unit of Albanian Nanoscience and Nanotechnology, 1000 Tirana, Albania

**Keywords:** 3-amino-1,2,4-triazole, diazonium salts, gold electrode, impedance spectroscopy, cyclic voltammetry, surface modification

## Abstract

Finding novel strategies for surface modification is of great interest in electrochemistry and material sciences. In this study, we present a strategy for modification of a gold electrode through covalent attachment of triazole (TA) groups. Triazole groups were electrochemically grafted at the surface of the electrode by a reduction of in situ generated triazolediazonium cations. The resulting grafted surface was characterized before and after the functionalization process by different electrochemical methods (cyclic voltammetry (CV), electrochemical impedance spectroscopy (EIS)) confirming the presence of the grafted layer. The grafting of TA on the electrode surface was confirmed using analysis of surface morphology (by atomic force microscopy), the thickness of the grafted layer (by ellipsometry) and its composition (by X-ray photoelectron spectroscopy). Density functional theory (DFT) calculations imply that the grafted triazole offers a stronger platform than the grafted aryl layers.

## 1. Introduction

The surface modification of materials is of immense importance, as it expands their general use. This enables one to add new characteristics to the grafted surface, including changes in wettability, adhesion, sensing properties, friction, wear, hardness, gloss, etc. In this regard, surface modification using aryl diazonium salts has become very popular. The concept was pioneered by Pinson and co-workers [1,2], and has proven to be a very versatile, simple, and fast process, compared to other conventional techniques, such as physical vapor deposition (PVD), chemical vapor deposition (CVD), ett). Electrochemical grafting has been investigated in detail over the last few decades [3,4], and has been applied to different types of materials [5,6], giving rise to a number of different applications, including sensor development [7,8], corrosion protection [9], and microelectronics [10].

**Scheme 1:**
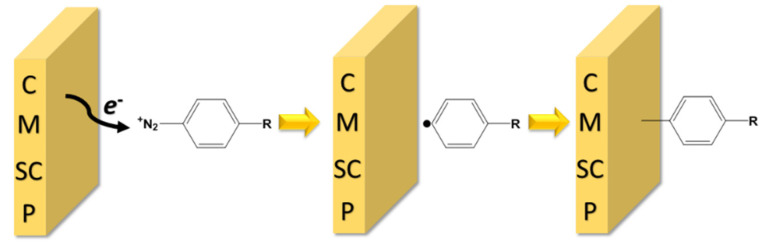
The mechanism of electrochemical grafting of diazonium to different substrates (C—carbon materials, M—metals, SC—semiconductors and P—polymers).

The electrode modification through covalent binding of the aryl radicals, derived from dediazotization reaction of the diazonium salts, is a simple process, which includes the formation of the corresponding radicals at the electrode vicinity and their grafting onto surfaces [5,11,12] (Scheme 1).

Although the aryl diazonium moiety can bear different functional groups, and post-modification reactions of the grafted layer are possible, finding new molecules that possess interesting chemical properties and the ability for surface grafting reactions is very important. Triazoles are widely known for their antibacterial properties. Our study aims to understand the grafting and the stability of such layers, as a first step towards the development of antibacterial surfaces.

In this study, 1,2,4-triazolediazonium is proposed for surface modification of the gold electrode. The triazolediazonium was synthesized in situ in a single step at ambient temperature, and used for grafting onto a gold electrode.

The surface of a gold electrode, before and after the modification, was characterized using electrochemical impedance spectroscopy (EIS) and cyclic voltammetry (CV) to confirm the presence of the grafted organic layer and its barrier property for the electron transfer. The chemical composition of the grafted surface was investigated by XPS, thereby proving the presence of triazole moieties. Additionally, the morphology and the thickness of the grafted surface were analyzed by atomic force microscopy (AFM) and ellipsometry measurements. Density functional theory (DFT) calculations show that the grafted triazole moiety is strongly bound to the interface, and is stronger compared to grafting using aryl [13] or alkyl [14] diazonium salts.

## 2. Materials and Methods

### 2.1. Reagents and Materials

Aqueous solutions were prepared using Milli-Q water (Q-POD’s Millipore system, Darmstadt, Germany). The following solvents and reagents were used as received: ethanol (EtOH, 99.9%), HCl (37% d = 1.18 g/cm^3^), and acetonitrile (ACN, 99.9%), HPLC grade, were purchased from Fisher Scientific (Bishop Meadow, Loughborough, UK). Potassium ferricyanide (K_3_[Fe(CN)_6_]) and potassium ferrocyanide (K_4_[Fe(CN)_6_]·3H_2_O), sodium nitrite (NaNO_2_, 99.8%), potassium chloride, tetrabutylammonium tetrafluoroborate (NBu_4_BF_4_), and 3-Amino-1,2,4 triazole were purchased from Sigma Aldrich (St. Louis, MO, USA).

The gold electrode (Au, 2.0 mm diameter) was purchased from CH Instruments, Inc. Austin, TX, USA.

Gold plate electrodes were ordered from Arrandee (Werther/Westfalen, Germany), specifically (2500 ± 500) Å thick gold film coated with a 25 ± 15 Å chromium layer on borosilicate glass support (1.1 × 1.1 cm).

### 2.2. Preparation of 3-diazonium. 1,2,4-triazole

The triazolediazonium salt (TAD) was prepared in situ through a diazotization reaction, following the procedure described by DeTraglia et Al. [15]. Briefly, 3-amino-1,2,4-triazole (ATA) (100 mg) was placed in an electrochemical cell and dissolved with HCl (1 M), to give a concentration of 1.19 mmol, with respect to the aminotriazole. The triazolediazonium salt solution was generated at room temperature in the electrochemical cell (in situ), by adding 82 mg of sodium nitrate (1.16 mmol) under vigorous stirring (5 min).

### 2.3. Preparation and Modification of Au Electrode

Before the surface modification, the gold electrode was polished with 1.0 μm alumina slurry (Buehler, Esslingen, Germany) and rinsed with Milli-Q water. Then, after sonication in an ultrasonic bath, with both ethanol and water (5 min each), the electrode was electrochemically cleaned in 0.5 M H_2_SO_4_ by running CV; 25 cycles, −0.3 to +1.5 V at 100 mV s^−1^ until a reproducible voltammogram was obtained. The cleaned electrode was finally rinsed with Mili-Q water.

The electrochemical modification of the Au-electrode surface was carried out using the concept of the in situ generation of TAD cations, as described in Section 2.4. For the generation of TAD, the solution was deaerated with ultra-pure nitrogen gas under vigorous stirring for 5 min, before immersion of the Au-electrode for electrografting [16]. The grafting of triazole (TA) to the gold surface was performed using chronoamperometry (E*_app_* = −0.55 V, t = 600 s). The potential (E_app_) was determined from cyclic voltammetry measurements (the reduction peak potential (E_red_).

The TA/Au electrode was rinsed with a copious amount of Milli-Q water, sonicated in acetonitrile for 180 s and rinsed again with Milli-Q water. Ultrasonication was performed to remove any physically adsorbed molecules that might remain on the Au electrode after grafting.

### 2.4. Instrumentation

#### 2.4.1. Electrochemical Measurements

All electrochemical measurements were carried out with a Modulab electrochemical system, ECS (Solartron Analytical, Farnborough, UK) and a conventional three-electrode system, containing a gold working electrode, an Ag/AgCl (saturated KCl) as reference electrode and a Pt wire as a counter electrode. Fe(CN)_6_^3−/4−^ (5 mM) in 100 mM KCl was used as a probe for the investigation of the Au-electrode, both before and after modification, using EIS and CV.

##### Electrochemical Impedance Spectroscopy

EIS was used for characterization of the Au-electrode, before and after grafting, using the Fe(CN) _6_^3−/4−^ redox probe. The EIS was carried out in the frequency range 65 kHz to 0.5 Hz with 10 data points/decade by applying an alternating voltage amplitude of 10 mV. The dc potential was always set up at 0.235 V, which is the standard potential of the redox probe—Fe(CN)_6_^3−/4−^. Experimental data of the electrochemical impedance plot were analyzed by Zview2 software (version 3.4 e)

##### Cyclic Voltammetry

CV was used both for characterization of the synthesized TAD and the modified electrode. The reduction of the generated TAD on Au electrode was checked by running CV from 0.4 to −0.6 V at 100 mV s^−1^. The Au-electrode was characterized, before and after modification, using the redox probe and scanning between +0.6 and −0.5 V at a scan rate of 100 mV s^−1^, to evaluate the barrier property of the grafted electrode for electron transfer.

#### 2.4.2. UV-VIS Spectroscopy

Varian Cary 50 Bio UV-visible Spectrophotometer was used for characterization of the in situ generated TAD and the corresponding parent amine within the range 200–400 nm.

#### 2.4.3. Atomic Force Microscopy (AFM)

The surface morphology of the TA-modified gold electrode, and that that of the bare gold electrode, was studied by AFM (Veeco, Plainview, NY, USA) with a NanoScope III fitted with a 125-m scanner (J-scanner) equipment with a scan size of 10.0 µm and scan rate 0.996 Hz under ambient conditions. Image processing and surface roughness calculations were performed via Nanoscope v720 software. The images were scanned in the topography, amplitude, and phase mode. Each AFM image presents numerous images taken on different sample areas. The thickness of the layer was determined by comparing the profile heights between the modified and unmodified areas.

#### 2.4.4. Ellipsometry

The film thickness and its refractive index was measured using an α - SETM ellipsometer (J.A. Woollam Co., Inc. (Lincoln, NE. USA). The measurements were performed at an incident angle of 70°.

Ellipsometry measures an alteration in polarization as light reflects or transmits from a material. The measured parameters are expressed as (Ψ) and (Δ), where (Δ) is the phase shift and tan (Ψ) is the amplitude ratio upon reflection. The complex refractive index of the bare substrate is found by using the measured parameters (Δ and Ψ). The real and the imaginary parts of the refractive index of the bare substrate were obtained by measuring the clean plates before modification.

Ellipsometric measurements were performed on the same area of the sample plates before and after electrografting.

The optical thickness was determined using a standard model available in the software (Complete Ease).

The B-Spline and the WvlByWvl models were used for the bare- and grafted gold electrodes, respectively.

#### 2.4.5. X-ray Photoelectron Spectroscopy (XPS)

The XPS spectra obtained on the grafted gold plates were collected with a Kratos Axis Ultra DLD electron spectrometer (Kratos Analytical, Manchester, UK). Monochromatic Al Kα source (hν = 1486.6 eV) was used at a power of 150 W. The survey scans were collected for binding energy spanning from 1100 eV to 0 with an analyzer pass energy of 160 eV. For the high-resolution spectra (N 1s, C 1s, and Au 4f), the pass-energy was 20 eV. The binding energy scale was set relative to the Au 4f_7/2_ binding energy at 84.0 eV. Krato software (version 2.2.9) was used for spectral analysis.

#### 2.4.6. Computational Details

DMol3 software (BIOVIA) is used for DFT calculations. The frequently employed gold cluster [13,14,17] model was used to evaluate the bond dissociation energy (BDE). Geometry optimization was achieved via the double numerical plus polarization basis set (DNP) [18] and the Perdew–Burke–Ernzerhof functional within the generalized gradient approximation (GGA–PBE) [19]. The van der Waals interactions were taken into account using the Tkatchenko-Scheffler (TS) method [20]. The lack of any imaginary frequencies ensured the energy minima of the optimized structures was reached [13,14]. For the calculations involving solvent (water or acetonitrile), the Conductor-like Screening Model (COSMO) was used [21]. The BDE was calculated as:

(1) $$BDE_{\left(Au13-TA\right)}=-\left(E_{Au13-TA}+E_{TA}+E_{Au13}\right)$$

where *E_Au13-TA_* is the total energy of the grafted Au13 cluster by a triazole moiety. *E_TA_* and *E_Au13_* are the energies of the isolated Au13 and TA entities [Equation (1)].

## 3. Results and Discussion

### 3.1. Characterization of Synthesized 3-diazonium-1,2,4-triazole Using UV

The initial examination of the in situ synthesized 3-diazonium-1,2,4-triazole was done using UV. A solution of 0.3 mM 3-diazonium-1,2,4-triazole was prepared by dilution of 3-diazonium-1,2,4-triazole into 1 mM HCl, and an examination of the absorption spectrum of 3-diazonium-1,2,4-triazole was made to confirm that diazotization had occurred.

**Chart 1:**
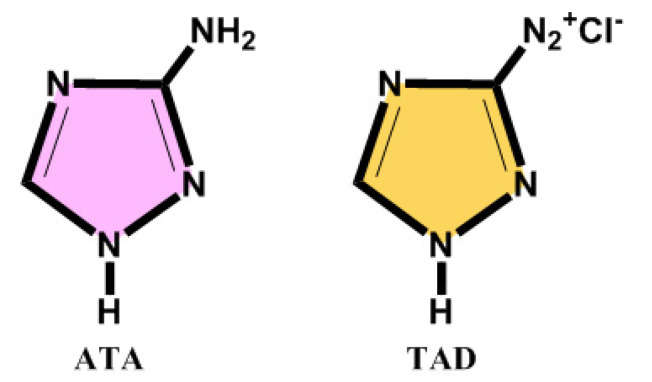
Chemical structures of 3-amino-1,2,4-triazole (ATA) and 3-diazonium-1,2,4-triazole (TAD).

The parent compound 3-amino-1,2,4-triazole (ATA) and the product 3-diazonium-1,2,4-triazole (TAD) have conjugated double bond systems (Chart 1) that influence the absorption peak wavelength and intensity.

**Figure 1:**
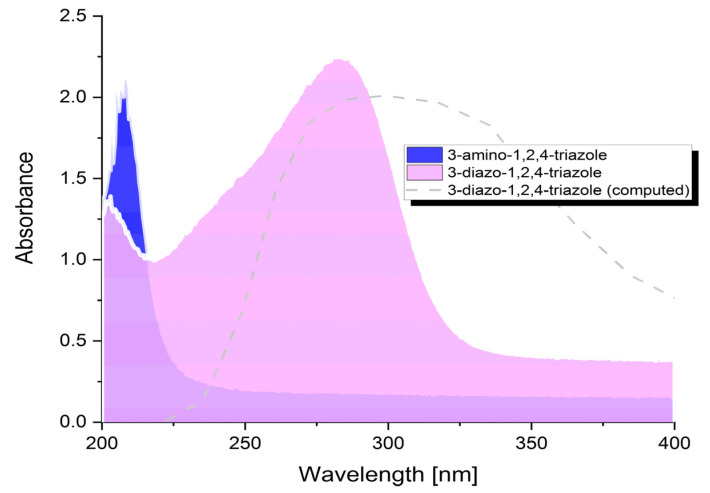
UV-VIS Absorption spectra of 3-amino-1,2,4-triazole and 3-diazonium-1,2,4-triazole.

Figure 1 shows the absorption spectra obtained from solutions of 0.3 mM of each ATA and TAD in 1 M HCl. The peak for TAD became bigger and appeared at a longer-wavelength (282 nm), compared to the parent compound (208 nm). The appearance of the absorption peak for the TAD at a longer wavelength can be ascribed to the increase in the size of the conjugated system brought up by the triple bond in N_2_. The observed differences in the absorption, between ATA and TAD, was also confirmed by simulations made using time-dependent density functional theory (TDDFT). The absorption for ATA at 199.2 nm (experimental value of (λmax. = 208.5 nm)) at small oscillator strength (f = 0.075) is dominated by the HOMO–LUMO transition. This excitation involves a slight shift of electron density from the NH_2_ nitrogen lone pair to the π system of the triazole ring. The absorption for TAD at the longest wavelength of 295.01 nm (experimental value of λmax. = 283.06 nm) at oscillator strength (f = 0.079) is dominated by the HOMO–LUMO transition. This excitation involves a slight shift of electron density from the π system of the triazole ring to the aligned antibonding π* LUMO orbital.

### 3.2. Characterization of in Situ Generated Triazolediazonium by Cyclic Voltammetry

The Au-electrode surface modification was performed following the procedure described in the experimental Section 2.3.

**Figure 2:**
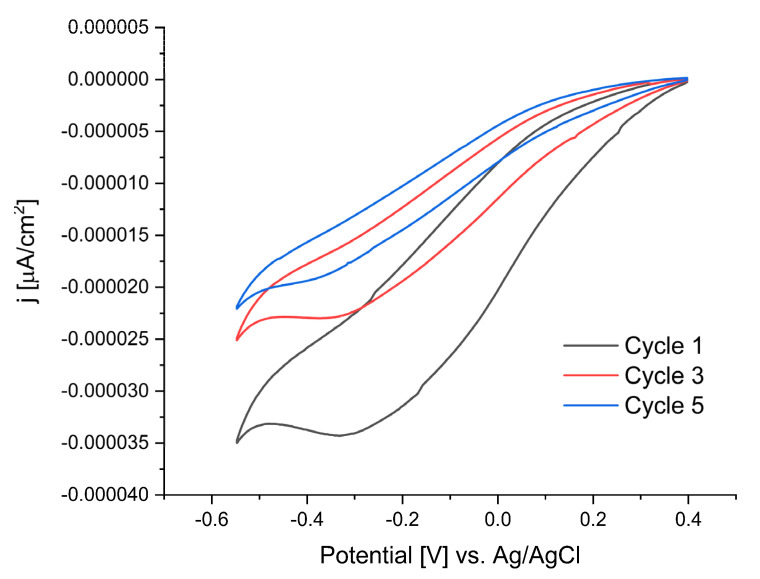
Cyclic voltammetry (CV) of (1.19 mmol) of 3-amino-1,2,4-triazole with (1.16 mmol) of NaNO_2_ in 5 ml 1 M HCl. Scan rate 100 mV and voltage range from +0.4 V-(−0.6 V). Cyclic voltammograms: black (first cycle), red (third cycle), blue (ffith cycle).

The cyclic voltammogram of TAD, scanned from 0.4 to −0.6 V, showed a reduction peak at ≈−0.3 V during the first cycle (Figure 2). The peak corresponds to the reduction of TAD to triazole radicals that subsequently bind on the gold surface. In the subsequent scans (only the third and fifth scans are shown in Figure 2) the peak current decreased, and the peak potential shifted to more negative values. The shift of the peak potential and decrease in current is due to the blocking of electron transfer, indicating that the surface is grafted during the preceding scan [1,5]. Similar behavior has been previously reported for the electrochemical reduction of several diazonium salts at both carbon [2,5,7] and gold electrodes [3,4,9,10,22,23].

**Figure 3:**
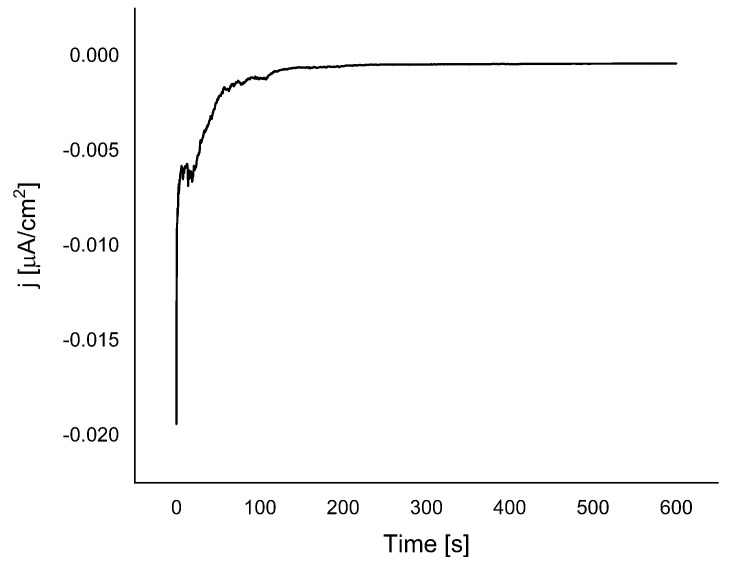
Chronoamperometry of (1.19 mmol) of 3-amino-1,2,4-triazole with (1.16 mmol) of NaNO_2_ in 5 ml 1 M HCl. Potential peak −0.55V and the reduction time 600 s.

The change of color of the grafted Au surface, after electrode modification using chronoamperometry, could also be seen by the naked eye. This may also suggest that a thick layer (visible) is formed when the electrode is grafted with TAD, which is not the case when grafting with the more known diazonium salts such as 4-carboxybenzediazonium. The chronoamperogram (Figure 3) shows the same known features (an exponential current decrease at the beginning of the grafting reaction ≈ up to 100 s, and a steady current due to the electrode blocking by the grafted layer), as in the case of the grafting of multilayer films from other aryl diazonium salts [11,24].

### 3.3. Characterization of the Electrode Modification Steps

#### 3.3.1. Cyclic Voltammetry with the Redox Probe, Fe(CN)_6_^3−/4−^

**Figure 4:**
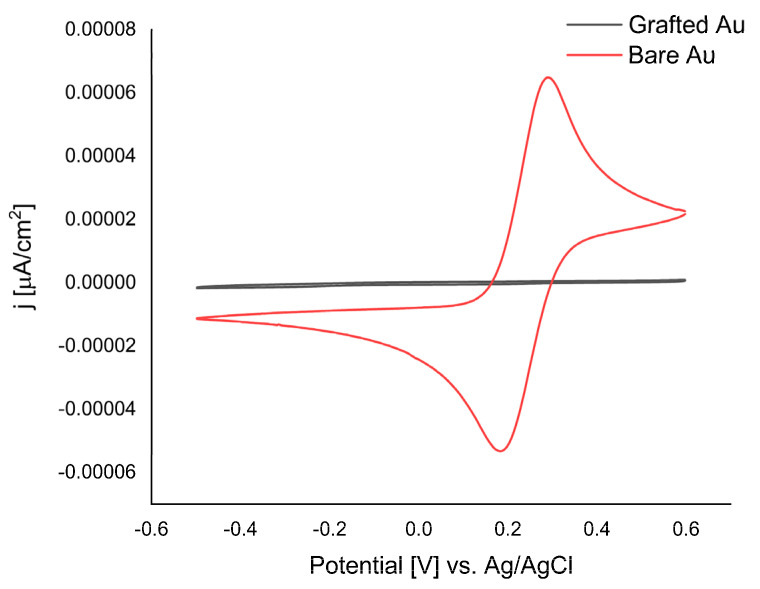
CV voltammograms recorded in ferricyanide/ferrocyanide (5.0 mM [Fe(CN)_6_]^3−/4−^ in 100 mM KCl) at bare Au and modified electrode using 3-diazo-1,2,4-triazole cation for 600 s at Ep −0.55 V.

The grafted electrode surface was characterized by cyclic voltammetry using Fe(CN)_6_^3−/4−^ as a probe. The voltammograms obtained for the probe before and after grafting the Au electrode are presented in Figure 4.

The bare Au electrode showed a quasi-reversible redox behavior, which is attributed to the reversible reduction and oxidation of the Fe(CN)_6_^3−/4−^. The redox peaks of Fe(CN)_6_^3−/4−^ disappeared for the triazole modified electrode. This reveals the grafting of the triazole layer on the electrode, leading to considerable blocking of the electron transfer.

A heterocyclic monolayer is not expected to reduce the Fe(CN)_6_^3−/4−^ electron transfer rate significantly. The formation of a multilayer should be possible, as it was evidenced by cyclic voltammograms. The blocking effect is significant, indicating that the grafting is quite efficient and the redox waves for the Fe(CN)_6_^3−/4−^ redox system almost disappear, a sign indicating that the gold surface is almost entirely covered. The possible formation of multilayers of TA was investigated using ellipsometry and AFM (see Section 3.4 and Section 3.5). The thickness of the layer is known to be dependent on concentration of the diazonium salt and a number of other variables, depending on whether CV or chronoamperometry is used for grafting. For CV, the variables are the number of cycles, the potential window, the scan rate; while for chronoamperometry they are applied potential and grafting time.

#### 3.3.2. Electrochemical Impedance Spectroscopy with the Redox Probe, Fe(CN)_6_^3−/4−^

The triazole layer grafted on the Au electrode was characterized using electrochemical impedance spectroscopy (EIS) in Fe(CN)63−/4−.

**Figure 5:**
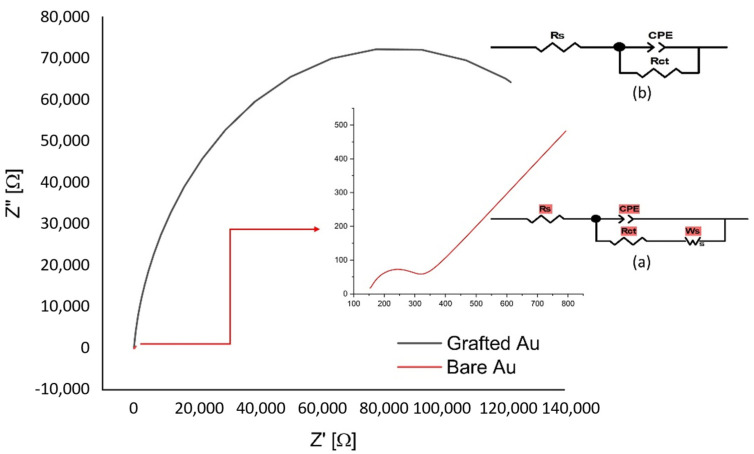
A. Electrochemical impedance spectroscopy (EIS) of 5.0 mM [Fe(CN)_6_]^3−/4−^ in 100 mM KCl, frequency range 65 kHz–0.5 Hz, ac-amplitude 10 mV and dc potential 0.235 V on bare (see the magnified inset) and electrode grafted with 3-diazo-1,2,4-triazole. B. Equivalent circuits used for the analysis of the electrochemical impedance data for the bare (**a**) and modified (**b**) electrodes.

Superimposed Nyquist plots for the bare and grafted gold electrodes are presented in Figure 5.

For clarity, the Nyquist plot for the bare electrode is put as an inset. The circuits used for fitting the impedance spectra are presented in Figure 5b.

The bare electrode shows a typical shape of a faradaic impedance spectrum for conductive electrodes, with a very small semicircle at high frequencies (Rct = 172 ohm), correlated to a very low electron transfer resistance, followed by a 45° straight line, which is typical of a diffusion-limited electron transfer process. The semicircle corresponds to a parallel blend of the charge transfer resistance Rct, with the double layer capacitance Cdl.

Data from EIS the charge–transfer resistance can be related to the electrode coverage, and is given by the following equation [25]:

(2) $$\left(1-\theta \right)=\frac{R_{CT}^{0}}{R_{CT}}$$

where θ is the electrode coverage, and $R_{CT}^{0}$ and *R_CT_* represent the charge transfer resistance measured on a bare and on a modified gold electrode.

The impedance plot for the grafted gold electrode differs significantly from that of the bare electrode. The diameter of the semicircle increased significantly (*R_CT_* = 1.69 × 10^5^ ohm), giving a surface coverage of 99.98% [Equation (2)]. This indicates that a compact blocking film is formed that slows down the redox reaction of the probe, as was also evidenced by CV (Figure 4).

The Warburg line was not observed for the modified electrodes in the frequency range 65 kHz to 0.5 Hz used here. The electron-transfer kinetics and the diffusional characteristics have been obtained by modelling the EIS spectra with a simple equivalent electrical circuit (see inset in Figure 5), for both bare and modified electrodes.

### 3.4. Ellipsometry

Ellipsometric measurements were performed on gold plates, before and after electrografting.

Since the measurements were carried out on a dried and therefore collapsed films, the refractive index of the layer was fixed at a constant value (real = 1.50; imaginary = 0), independent of the thickness. The thickness of the deposited layer on the gold electrode surface, calculated from the average measurements done on five different spots was found to be 24.7 ± 0.3 nm. The layer thickness data obtained from ellipsometry measurements correlated well with AFM (Section 3.5).

### 3.5. Surface Characterization of TA Modified Au Electrodes by AFM

**Figure 6:**
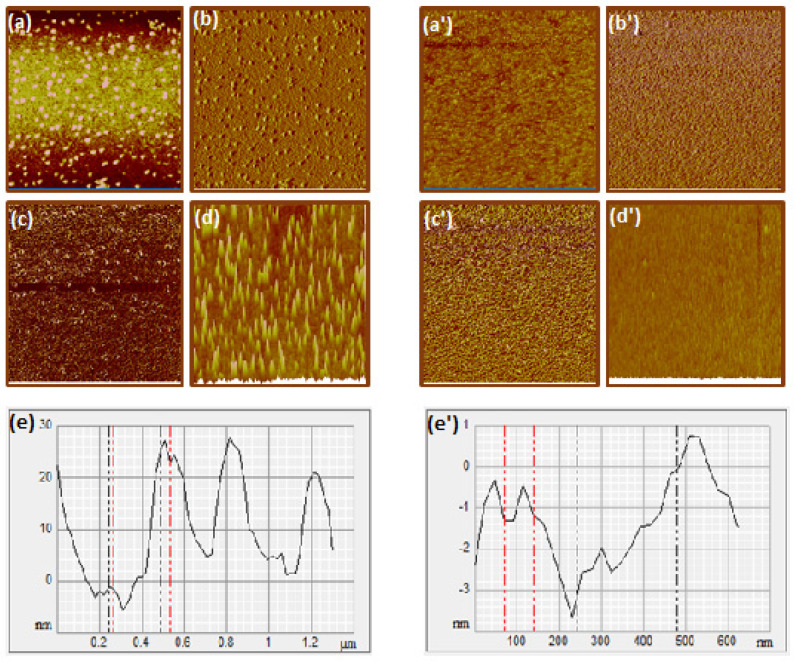
A. Atomic force microscopy (AFM) images (**a,a’**) topographic; (**b,b’**) phase; (**c,c’**) amplitude (**d,d’**) 3D surface (**e,e’**) roughness profile (**a**–**e**—modified Au electrode) and (**a’**–**e’**—unmodified Au electrode).

The AFM images in Figure 6 show a clear difference in the morphology of the grafted and bare gold surfaces.

The AFM images of heterocyclic-modified electrodes show clearly that the surfaces of the electrodes are fully covered with triazole layer. The granular features of the electrode modified by chronoamperometry 600 s at potential (−0.55 V) were uniform with a (24.2 ± 0.2 nm) average diameter, which is in good agreement with the ellipsometric measurements (~24.7 ± 0.3 nm).

These results indicate that the electrochemical process yields films that are much thicker than one monolayer (geometrical model of a single TA molecule is ~0.35 nm) supporting the CV results suggested from the redox probe measurements. This suggests a multilayer film composed of at least 70 TA moieties.

### 3.6. Characterization of Au-TA by XPS

For the bare gold surface, the survey spectrum gave the expected main peak at the binding energy of 84 eV for gold (Au4f7/2). The binding energies of 284.2 eV and 531.6 eV correspond to trace amounts of carbon (C 1s) and oxygen (O 1s), respectively, which is probably due to the adsorption of organic contaminants during and after the cleaning process in a basic Piranha solution.

After grafting of the TA on the gold surface, the intensities of the Au and oxygen peaks decreased significantly, whereas those of carbon and nitrogen increased dramatically. A significant decrease of the Au4f peak upon surface modification with TA groups can be due to the formation of a thick film (multilayers) that mask the signal of Au4f7/2 [24].

The N 1s core level spectrum for the triazole modified electrode that accounted for 45.04% can be deconvoluted to two contributions. The main peak at 399.5 eV, which accounted for 29.83% of the total nitrogen, is attributed to the neutral nitrogen in the aromatic ring of 1.2.4-triazole (=N-) [26,27,28] and the peak at 401.3 eV, representing 15.21% of the total nitrogen, corresponds to protonated or hydrogen-bonded nitrogen (N-H) of the triazole moiety [29].

The ratio between the peak percentages of (=N-) and (N-H), 29.83:15.21 (≈2:1) is in good agreement with the structure of the TA. This provides direct evidence for the attachment of the triazole ring on the surface of the Au electrode. Subsequently, we need to account for the precursor diazonium cations. The absence of peaks at binding energies of 403.7 and 405.6 eV that correspond to N≡N is also another indication that the N_2_ in TAD is cleaved, producing a radical that covalently binds to the gold surface.

The C 1s spectra showed a main peak at 286.8 eV corresponding to (C=N)(C-N) bonds in the triazole ring [26,27] (28.17% of the total carbon), and another peak at 288.2 eV for (C-NH) [30] (2.19% of the total carbon). The total atomic percentage of carbons on the ring is thus 30.36%.

The ratio of nitrogen/carbon in the TA is 3 to 2, which is in accordance with the atomic percentage of 45.04% N to 30.36% C obtained from XPS. These peaks confirm the grafting of the TAD on the gold electrode surface.

A more detailed analysis of the surface composition is summarized in Table 1, for the grafted Au electrode at atomic surface concentrations (at.%) obtained by the integration of the core level peaks.

### 3.7. DFT

**Figure 7:**
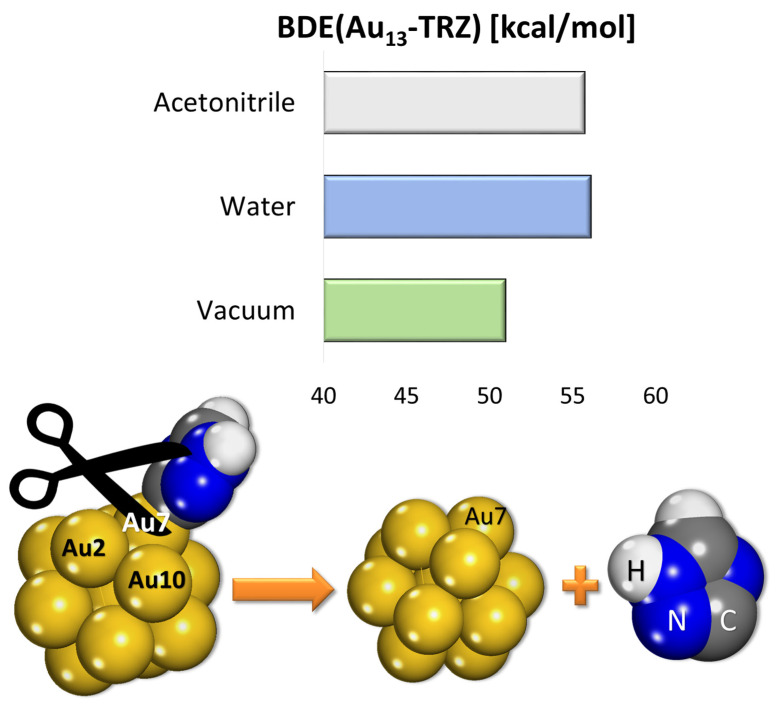
Bond dissociation energy (BDE) for the Au13-TA grafted cluster and the scheme for the BDE calculations (optimized structures).

The bond dissociation energy (BDE) between the Au13 gold cluster and the grafted triazole moiety in a vacuum, water and acetonitrile, together with the BDE scheme (optimized geometry in the vacuum) is presented in Figure 7.

The grafted triazole layer has a BDE of ≈56 kcal/mol (in solvent) or ≈51 kcal/mol. In the literature, the binding energy of grafted phenyl or alkyl moieties varies between 34.4 to 36.9 kcal/mol [13,14]. These results point out to a stronger interface, compared to the grafted alkyl or radical and even to thiol monolayers (as self-assembled monolayers, which are frequently used for the surface modification of gold) [31]. The attachment of triazole moiety onto the Au13 surface influences some geometry changes, in line with previous observations [13]. The bond distances between neighboring gold atoms is altered after the grafting: in the bare cluster d(Au2-Au7) = 3.069 Å, d(Au10-Au7) = 3.064 Å, while after grafting to Au7 d(Au2-Au7) = 3.073 Å, d(Au10-Au7) = 3.084 Å, demonstrating a similar effect to that evidenced in the case of grafted phenyl moiety [13].

## 4. Conclusions

This work shows, for the first time, the electrografting of in situ synthesized 1,2,4-triazolediazonium (TAD) on a gold surface.

Studies made on the grafted 1,2,4-triazole (TA) layer by EIS and CV, using Fe(CN)_6_^3−/4−^ as a redox probe, revealed the blocking properties of the TA for the electron transfer. The surface coverage (99.98%), was calculated from the EIS data.

The AFM and ellipsometry measurements displayed that the film is formed as a multilayer. The main functional groups of the TA grafted on the surface could be confirmed by XPS. Theoretical calculations showed that the BDE value of the grafted triazole moiety is larger, compared to other molecules (thiols, alkyl or aryl moieties derived from corresponding diazonium salts). This indicates that the covalent binding of the TA to the Au surface is more stable, opening several potential applications, ranging from sensors to the corrosion protection of materials.

## Figures and Tables

**Table 1:** Atomic concentrations (at.%) of different elements at the Au electrode surface after the modification step by the in situ method are reported.

Line	BE, eV	AC, at.%
N 1s	399.5	29.83%
	401.3	15.21%
C 1s	286.8	28.17%
	288.2	2.19%
O 1s	531.8; 533.2	3.77%
